# Supplementary material for: ProteinShader: illustrative rendering of macromolecules
Source: BMC Struct Biol. 2009 Mar 30;9:19. doi: 10.1186/1472-6807-9-19 (PMC2672931; doi:10.1186/1472-6807-9-19)
Supplement: Additional file 1 — ProteinShader program without source code. This compressed file contains the complete ProteinShader program including associated libraries, but no source code. A README.txt file gives an overview of the ProteinShader distribution, and the index.html file in the help subdirectory has directions on getting started with the program as well as a set of tutorials. [file 1472-6807-9-19-S1.zip › ProteinShader-beta-0_9_4-binary/help/api/org/proteinshader/graphics/typography/package-frame.html]

org.proteinshader.graphics.typography (ProteinShader API)


org.proteinshader.graphics.typography

Interfaces 
  
*GlyphSetFactory*

Classes 
  
GLF2GlyphSetFactory
  
GLF2Header
  
Glyph
  
GlyphDemo
  
GlyphSet
  
GlyphSetFactoryFactory
  
TextLabel
  
TextLabelFactory
  
TextLabelManager
